# Constituents of Saffron (*Crocus sativus* L.) as Potential Candidates for the Treatment of Anxiety Disorders and Schizophrenia

**DOI:** 10.3390/molecules21030303

**Published:** 2016-03-02

**Authors:** Nikolaos Pitsikas

**Affiliations:** Department of Pharmacology, School of Medicine, Faculty of Health Sciences, University of Thessaly, Panepistimiou 3 (Biopolis), Larissa 41500, Greece; npitsikas@med.uth.gr; Tel.: +30-2410-685-535

**Keywords:** *Crocus sativus* L., anxiety, stress, schizophrenia

## Abstract

Anxiety disorders and schizophrenia are common public health issues. The dried stigma of the plant *Crocus sativus* L., (*C. sativus*) commonly known as saffron are used in folk medicine for various purposes. Several lines of evidence suggest that *C. sativus*, crocins and safranal are implicated in anxiety and schizophrenia. Here, I intend to critically review advances in research of these emerging molecules for the treatment of anxiety and schizophrenia, discuss their advantages over currently used anxiolytics and neuroleptics, as well remaining challenges. Current analysis shows that *C. sativus* and its components might be a promising class of compounds for the treatment of the above mentioned psychiatric diseases.

## 1. Introduction

*Crocus sativus* L. (*C. sativus*), is a perennial herb member of the Iridaceae family, the line of Liliaceae. This plant is cultivated in many countries such as Azerbaijan, China, France, Greece, Egypt, India, Iran, Israel, Italy, Mexico, Morocco, Spain and Turkey. Its product is the well-known spice called saffron. Saffron, in filaments, is the dried dark-red stigmas of *C. sativus* flower [1]. One stigma of saffron weighs about 2 mg and each flower has three stigmata; 150,000 flowers must be carefully picked one by one to obtain 1 kg of spice. Saffron has a distinct colour, flavour and odour. It is used both as a spice for flavouring and colouring food preparations, and as a perfume. The stigmas of it are also used in folk medicine as an anticatarrhal, eupeptic, antispasmodic, expectorant, emmenagogue and nerve sedative (for review see [2]). Further, saffron has widely been used in Persian traditional medicine for memory problems [3]. The present review was designed to critically assess the role played by *C. sativus* and its active constituents in anxiety and schizophrenia since the current pharmacotherapy for both these diseases is not satisfactory.

## 2. Chemistry of *C. sativus*

Chemical analysis of *C. sativus* stigmas has shown the presence of about 150 volatile and non-volatile compounds. Fewer than 50 constituents, however, have been identified so far [4]. The volatiles consist of more than 34 components that are terpenes, terpene alcohols and their esters among which safranal is the main component. Non-volatile compounds comprise crocins, crocetin, picrocrocin and flavonoids (quercetin and kaempferol) [5].

**Figure 1:**
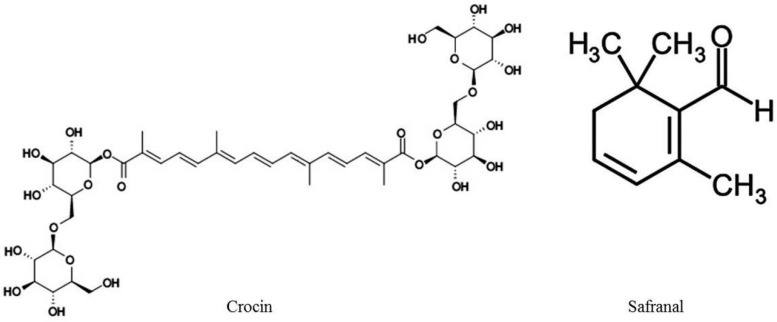
Molecular structures of *C. sativus* components crocin and safranal.

In particular, crocins, glucosyl esters of crocetin, are water-soluble carotenoids and are responsible for saffron’s characteristic colour. Picrocrocin, glycoside of safranal, is responsible for the bitter taste of the spice and is the precursor of safranal. Safranal, the main component of the distilled essential oil, is a monoterpene aldehyde, responsible for its characteristic aroma [6,7]. In Figure 1 the molecular structure of C. *sativus* components crocin and saffranal is illustrated.

## 3. Effects of *C. sativus* and Its Constituents on Anxiety

Anxiety may be interpreted as an emotional anticipation of an aversive situation and is reflected by species-specific behavioural fear responses to stressful and threatening stimuli, characteristic for individual trait anxiety. Anxiety disorders including generalized anxiety disorder (GAD), specific and social phobias, post-traumatic stress disorder (PTSD), obsessive-compulsive disorder (OCD) and panic disorder are a major public health issue worldwide.

To date, anxiety disorders have been treated with medications that target γ-aminobutyric acid (GABA) and serotonergic neurotransmission, like benzodiazepines, partial agonists of the serotonergic 5-HT_1A_ receptor and selective serotonin re-uptake inhibitors (SSRIs). Some forms of anxiety, however, are relatively resistant to treatment with these agents [8,9]. In addition, either benzodiazepines or SSRIs can be associated with severe side effects, such as sedation, memory deficits, dependence and withdrawal, sexual dysfunction and weight gain. Further, the 5-HT_1A_ receptor partial agonist buspirone has a somewhat limited use. Although it is generally well tolerated with few side effects, it has lower efficacy and its onset of action is slower than previous drugs such as the benzodiazepines [10]. Thus, there is an urgent need to develop alternative treatment strategies [11].

### 3.1. Preclinical Studies

An overview of the preclinical literature regarding the effects of *C. sativus* and its constituents on anxiety is provided in Table 1. Experimental evidence indicated that crocins displayed an anxiolytic-like effect in procedures assessing anxiety in rats [12,13]. Particularly, in the light/dark test, administration of 50 mg/kg crocins, similarly to the reference compound diazepam (1.5 mg/kg), increased the time to enter the dark compartment, did not affect the number of transitions between the light and the dark chamber of the apparatus and prolonged the time spent in the lit compartment of the light/dark box [12]. In addition, crocins (30 and 50 mg/kg) were found to reduce compulsive behaviour (excessive grooming) induced by the serotonergic 5-HT_2c_ receptor agonist 1-(3-chlorophenyl)piperazine hydrochloride (mCPP) (0.6 mg/kg) [13]. Interestingly, crocins, at any dose tested, did not alter rats’ locomotor activity. Collectively, the results of the above described studies suggest that these active constituents of *C. sativus* induce anxiolytic-like behaviour in the rat and their anxiolytic effects cannot be attributed to changes in locomotor activity [12,13].

In a study carried out in mice, it has been revealed that low doses of the aqueous extracts of saffron (56 and 80 mg/kg) and safranal (0.15 and 0.35 mL/kg) induced an anxiolytic-like effect not different from that of diazepam (3 mg/kg) because they increased the time spent in the open arms of an elevated plus maze. At higher doses (320 and 560 mg/kg), the aqueous extracts of saffron did not display any anti-anxiety effect. It is important to underline that the aqueous extracts of saffron, at any dose tested, produced sedation since they reduced mice’ motility and motor coordination evidenced in the open field and rotarod tests, respectively. Safranal at a low dose range (0.05 and 0.15 mL/kg) also induced hypomotility and increased grooming activity and rearing. Crocin (50–600 mg/kg) did not affect mice behaviour in the elevated plus maze test, and at 200 and 600 mg/kg reduced mice motility [14].

The results of this latter study [14] appear to be in contrast with the above described findings [12,13] in which an anxiolytic effect of crocins has been observed. These discrepant findings may be attributable to differences in experimental settings (type of animal, pharmacological design, dose range, behavioural procedure).

Finally, administration of aqueous extracts of saffron (1–10 mg/kg), of crocin (1–10 mg/kg) but not of ethanolic extracts of *C. sativus* (1–10 mg/kg) and safranal (1–10 mg/kg) reduced stress-induced anorexia in the mouse and did not influence plasma corticosterone levels. These results suggest an anti-stress effect of saffron and crocin [15].

### 3.2. Mechanism of Action of C. sativus and Its Constituents in Anxiety Disorders

The precise mechanism(s) by which saffron and its active components produced their anxiolytic effects, which are of the same magnitude of those produced by the benzodiazepine diazepam, is still under investigation. Benzodiazepines are compounds that produce their anxiolytic effect by acting as indirect agonists on the GABA_A_ receptor. In this context, it has been shown that some flavonoids isolated from plants display an affinity for the benzodiazepine binding site at the GABA_A_ receptor [17,18]. It can be hypothesized that saffron and its components, similarly to other flavonoids, exert their anxiolytic action by interacting with the benzodiazepine binding site at the GABA_A_ receptor. Additional studies are required, however, to address this issue.

The pharmacological mechanism(s) that might account for the potential anti-stress effects of *C. sativus* and its components has not yet been clarified. Several lines of evidence indicate that stress activates the hypothalamus-pituitary-adrenal (HPA) axis which leads to the plasma corticosterone increment as a response [19]. According to recent findings [15], mice that received saffron aqueous extracts or crocin did not show elevation of plasmatic corticosterone levels under stress. It is likely that crocin may interact with the HPA axis and reduce the stress-induced corticosterone increase [15]. In line with the above results, it has been demonstrated that saffron can inhibit *N*-methyl-d-aspartate (NMDA) and sigma, St. Louis, MO, USA, opioid receptors [20]. The latter is of importance since NMDA and sigma receptors can regulate corticosterone release from the adrenal cortex in rats [21]. It can be concluded that saffron and crocin may inhibit corticosterone secretion in stressed mice via blockade of NMDA and/or sigma opioid receptors located in the adrenal cortex [15].

## 4. Effects of *C. sativus* and Its Constituents in Schizophrenia

Schizophrenia is a serious mental disorder that affects up to 1% of the population worldwide. It is a complex heterogeneous syndrome which impairs social, occupational and individual functioning and results in a remarkable decline in the quality of life of patients. Its aetiology and pathophysiology remain unknown. Schizophrenic patients suffer from enduring and persistent psychotic symptoms, which can be divided in three major types: positive symptoms (f.i., hallucinations, delusions, disordered thought processing, catatonic behaviour), negative symptoms (social withdrawal, anhedonia, avolition) and cognitive disturbances (deficits in attention and memory) [22].

Abnormalities in a number of neurotransmitter systems, most notably the dopamine, glutamate, cholinergic, the serotonergic and the GABAergic systems, are thought to be important for the appearance of this disease [23]. In particular, positive symptoms of schizophrenia are associated with an excess of dopaminergic neurotransmission, in striatal brain regions, while negative symptoms and cognitive deficits are linked to dopaminergic hypofunction in prefrontal brain regions.

Moreover, consistent experimental evidence proposes a role for glutamate hypofunction in the pathophysiology of schizophrenia. NMDA receptor dysfunction is linked to secondary dopaminergic dysregulation in striatal and prefrontal brain regions. In addition, clinical observations have demonstrated that pharmacological blockade of NMDA receptor produced the component symptoms-negative symptoms and cognitive impairment that were neither affected by antipsychotics nor produced by dopaminergic agonists [24]. Further, inhibitory GABAergic neurotransmission appears to be impaired in schizophrenia patients [25]. In this context, it is important to underline that GABAergic firing regulates dopamine transmission in the prefrontal cortex and a GABA interneuron deficit in schizophrenia has been proposed to underlie some of the clinical symptoms [26].

Although traditional antipsychotic drugs have demonstrated utility in treating the positive symptoms of schizophrenia, current treatments are limited in their ability to alleviate the negative and cognitive symptoms’ clusters and often are accompanied by significant side effects which themselves impact the quality of life [27]. Finally, one third of patients are resistant to currently available medication. Therefore, there is an urgent requirement to develop new molecules for the treatment of schizophrenia.

### 4.1. Preclinical Studies

An overview of the existing literature regarding the effects of *C. sativus* and its constituents on schizophrenia is provided in Table 1. Acute administration of crocins (15–30 mg/kg) reversed recognition memory deficits produced by the NMDA receptor antagonist ketamine (3 mg/kg) in rats eliciting, thus, the effects of this active constituent of *C. sativus* in schizophrenia-related cognitive deficits. In addition, crocins (50 mg/kg) attenuated ketamine (25 mg/kg)-induced psychotomimetic effects (hypermotility, stereotypies and ataxia) in the rat. Further, using the social interaction test, a procedure resembling the negative symptoms of schizophrenia, these active constituents of saffron (50 mg/kg) were found to attenuate the social isolation induced by sub-chronic treatment with ketamine (8 mg/kg) in rats [16].

### 4.2. Clinical Studies

Up to now, only one clinical trial has been performed aiming to assess the safety and tolerability but not the efficacy of saffron extracts and crocin in schizophrenia. This double-blind placebo-controlled study was carried out in 61 schizophrenia patients. Schizophrenics received treatment twice daily (saffron or crocin 15 mg) or placebo for 12 consecutive weeks. In line with prior findings [28,29,30], the results of this study showed that saffron extracts and crocin administered at 15 mg twice daily were safe and well tolerated in schizophrenic patients [31].

### 4.3. Mechanism of Action of *C. sativus* and Its Constituents in Schizophrenia

The mechanism(s) through which crocins exert their effects on ketamine-induced behavioural deficits are not yet clarified. Additional studies should be carried out aiming to elucidate this issue. It is well documented, however, that schizophrenia-like effects of NMDA receptor antagonists (e.g., ketamine) include increased levels of glutamate, hypermotility, stereotypy and cognition deficits [32]. In this context, it has been reported that acute systemic administration of safranal reduced kainic acid-induced increase of extracellular glutamate concentrations in the rat hippocampus [33]. Further, it has been demonstrated that *C. sativus* extracts inhibited glutamatergic synaptic transmission in rat cortical brain slices [34]. Collectively, these findings suggest that this reduction of glutamate levels by saffron and its constituents might be critical for the beneficial action exerted by crocins on ketamine-induced behavioural deficits.

An alternative hypothesis to explain the beneficial action of crocins in an animal model of schizophrenia is based on the well-known antioxidant properties of crocins [35,36,37,38]. In agreement with the above studies it has been reported that saffron extracts and crocins conferred protection against oxidative stress and spatial learning deficits induced by chronic stress in rats [39]. Although the pathogenesis of schizophrenia remains unknown, a possible relationship between oxidative stress and the disease has been proposed [40], and sub-anesthetic doses of ketamine have been reported to increase oxidative stress in rats’ brain [41]. Specifically, following treatment with different sub-anesthetic doses of ketamine, an increase in oxidative damage marked by an increase in lipid peroxidation, oxidative protein damage and a decrease in enzymatic defense was observed in an animal model of schizophrenia [41]. As a whole, these data indicate that the beneficial effects of crocins on ketamine-induced behavioural deficits might be associated with their antioxidant properties.

## 5. Effects of *C. sativus* and Its Constituents on Other Neurological/Neuropsychiatric Diseases

### 5.1. Anticonvulsant Activity, Neurodegeneration

Studies performed in rodents revealed a certain anticonvulsant activity of aqueous and ethanolic extracts of *C. sativus* and its active component safranal [42,43]. Saffron and its active constituents affect a number of different neural processes. These molecules conferred protection in a rat model of Parkinson disease (PD) [44] and in animal models of cerebral ischemia [45,46,47].

### 5.2. Memory

Accumulating evidence indicates that *C. sativus* and its major component crocin are significantly involved in cognition. Preclinical studies demonstrated their efficacy in attenuating memory disorders in animal models related to Alzheimer disease (AD), cerebral injuries or schizophrenia (for review see [48]).

Clinical research has evaluated the efficacy of saffron in humans suffering from memory problems as are the Alzheimer disease (AD) patients. The results of clinical studies indicate that the effects exerted by ethanolic extracts of saffron on cognition were not different than those expressed by the reference compounds donepezil and memantine in reducing the cognitive decline in patients with mild-to-moderate and moderate to severe AD. In this context, it is important to emphasize the good safety profile of saffron which was revealed in all clinical studies [28,29,30].

### 5.3. Depression

*C. sativus* and its active components crocin and safranal have shown antidepressant-like effects in animal models of depression [49]. Importantly, clinical research findings reinforced preclinical results and proposed that saffron is efficacious for the treatment of mild-to-moderate depression [50,51]. Interestingly, it has been demonstrated that saffron antagonized sexual dysfunction in humans induced by the SSRI fluoxetine which is the widest used antidepressant in our days [52,53].

## 6. Effects of *C. sativus* and Its Constituents on Other Non-Neurological/Neuropsychiatric Diseases

Preclinical pharmacological studies have demonstrated that *C. sativus* crude extracts and purified chemicals possess anti-tumour effects (e.g., [54,55,56]). Saffron and its ingredients display antinociceptive, and anti-inflammatory properties [57], reduce atherosclerosis [58] and hepatic damage [59], counteract hyperlipidaemia [60], provide protection from myocardial injury [61] and display antihypertensive action [62,63]. The effects exerted by saffron and its components in the aforementioned pathologies were also extensively discussed in different reviews (e.g., [2,64,65,66]).

The outcome of these preclinical studies indicates that *C. sativus* and its components exert a beneficial action in several pathologies. It is important to underline, however, that up to now there has been no clinical information on the potential efficacy of saffron and its constituents in the aforementioned pathologies. Future clinical research should address this issue.

## 7. Safety Evaluation of *C. sativus* and Its Constituents

Toxicity studies have demonstrated that the hematological and the biochemical parameters were within a normal range in mice treated with saffron extracts [54]. It has also been reported that the oral LD_50_ of saffron was 20.7 g/kg administered as a decoction in mice [67]. Further, a recent work investigated either the acute (up to 3 g, both orally (p.o.) and intraperitoneally (i.p.)) or the sub-chronic effects of crocin (15–180 mg/kg, i.p.) in different biochemical, hematological and pathological parameters in rodents. The results of this study demonstrated that chronic treatment with crocin did not alter the weight of heart, lung, liver, kidney and spleen. Crocin, at the highest dose (180 mg/kg), increased platelets and creatinine levels, and reduced food intake and body weight. A decline in alveolar size in lungs was observed following the highest dose of crocin (180 mg/kg). The authors concluded that crocin, at pharmacological doses, was not shown to markedly damage any of the major organs of the body [68].

Interestingly, the findings of clinical studies suggest that both *C. sativus* extracts and crocin display a relatively safe and normal pharmacological profile. Specifically, in a double-blind, placebo-controlled trial conducted among healthy volunteers, a one-week treatment with saffron (200–400 mg/day) did not evidence particular alterations [69]. The results of another double-blind, placebo-controlled study performed in healthy volunteers also showed that administration for one month of crocin (20 mg/day) did not elicit significant alterations of different hematological, biochemical, hormonal and urinary parameters recorded [70].

## 8. Conclusions

There is scant experimental evidence, either preclinical or clinical, regarding the involvement of *C. sativus* and some of its active constituents in anxiety and schizophrenia. In spite of it, the few preclinical results are of certain consistency. Several issues, however, have not been addressed at all. There is no information on the potential efficacy of saffron in other anxiety disorders such as GAD, social phobia, panic and PTSD. There is also poor information regarding the potential antipsychotic action of saffron. There is no experimental evidence whether these compounds can counteract attentional deficits which are considered as a prominent aspect of cognitive dysfunction in schizophrenia. Additional research, using genuine animal behavioural models, is mandatory to establish whether these molecules might be a potential therapeutic tool for the treatment of anxiety disorders and schizophrenia. In this context, it is important to emphasize the good safety profile of saffron which was revealed in all preclinical and clinical studies here presented.

## Figures and Tables

**Table 1:** Effects of *Crocus sativus* L., and its constituents on animal models of anxiety disorders and schizophrenia.

Species	Agent	Dose Range	Route	Behavioural Test	Effect	Ref.
Rat	Crocins	15, 30, 50 mg/kg	i.p. acute	Light/dark	Anxiolytic-like (50 mg/kg) effect	[12]
				Motor activity	No effect	
Mouse	CsAE	56, 80, 320, 560 mg/kg	i.p. acute	Elevated plus maze	CsAE (56, 80 mg/kg), safranal (0.15, 0.35 mL/kg) anxiolytic effect. Crocin was ineffective.	[14]
	Crocin	50, 200, 600 mg/kg	i.p. acute	Open field	CsAE (dose-dependently), crocin (200–600 mg/kg), reduced motility, grooming, rearing, leaning.	
	Safranal	0.05, 0.15, 0.35 mL/kg	i.p. acute	Rotarod	CsAE (dose-dependently), crocin (200–600 mg/kg), reduced motility, grooming, rearing, leaning.	
					Safranal (0.05, 0.15 mL/kg) reduced motility; (0.15, 0.35 mL/kg) increased grooming, leaning, rearing.	
					CsAE (dose-dependently) decreased motor coordination. Crocin and safranal were ineffective.	
Mouse	CsAE	1, 5, 10 mg/kg	i.p. acute	Food intake	CsAE and crocin decreased stress-induced anorexia.	[15]
					Plasma corticosterone levels were not increased in CsAE and crocin-treated mice.	
	CsEE	1, 5, 10 mg/kg	i.p. acute		CsEE and safranal were ineffective.	
	Crocin	1, 5, 10 mg/kg	i.p. acute			
	Safranal	1, 5, 10 mg/kg	i.p. acute			
Rat	Crocins	15, 30 mg/kg	i.p. acute	Measurement of grooming behaviour	Attenuated mCPP-induced excessive grooming (anxiolytic effect).	[13]
	m-CPP	0.6 mg/kg	i.p. acute.	Motor activity	No effect	
Rat	Crocins	15, 30, 50 mg/kg	i.p. acute	NORT	Crocins (15, 30 mg/kg) counteracted ketamine-induced recognition memory deficits.	[16]
	Ketamine	3 mg/kg (NORT)	i.p. acute	NORT	Crocins (15, 30 mg/kg) counteracted ketamine-induced recognition memory deficits.	
				SI	Crocins (50 mg/kg) attenuated ketamine-induced social isolation.	
	Ketamine	8 mg/kg (SI)	i.p. sub-chronic	SI	Crocins (50 mg/kg) attenuated ketamine-induced social isolation.	
				Motor activity, stereotypies, ataxia	Crocins (50 mg/kg) attenuated ketamine-induced hypermotility, stereotypies and ataxia.	
	Ketamine	25 mg/kg (motor activity)	i.p. acute	Motor activity, stereotypies, ataxia	Crocins (50 mg/kg) attenuated ketamine-induced hypermotility, stereotypies and ataxia.	

CsAE, *Crocus sativus* Aqueous Extracts; CsEE, *Crocus sativus* Ethanolic Extracts; i.p., intraperitoneally; mCPP, 1-(3-cholorophenyl)piperazine; NORT, novel object recognition task; SI, social interaction.
